# Distribution of Immune Cells Including Macrophages in the Human Cochlea

**DOI:** 10.3389/fneur.2021.781702

**Published:** 2021-11-22

**Authors:** Wei Liu, Niklas Danckwardt-Lillieström, Anneliese Schrott-Fischer, Rudolf Glueckert, Helge Rask-Andersen

**Affiliations:** ^1^Department of Surgical Sciences, Section of Otorhinolaryngology and Head and Neck Surgery, Uppsala University, Uppsala, Sweden; ^2^Inner Ear Laboratory, Department of Otorhinolaryngology, Medical University Innsbruck, Innsbruck, Austria

**Keywords:** human, cochlea, macrophages, CX3CL1 genes, RNA *in situ* hybridization

## Abstract

**Background:** The human cochlea was earlier believed to lack capacity to mount specific immune responses. Recent studies established that the human cochlea holds macrophages. The cells appear to surveil, dispose of, and restore wasted cells to maintain tissue integrity. Macrophage activities are believed to be the central elements in immune responses and could swiftly defuse invading microbes that enter via adjacent infection-prone areas. This review updates recent human studies in light of the current literature and adds information about chemokine gene expression.

**Materials and Methods:** We analyzed surgically obtained human tissue using immunohistochemistry, confocal microscopy, and multichannel super-resolution structured illumination microscopy. The samples were considered representative of steady-state conditions. Antibodies against the ionized calcium-binding adaptor molecule 1 were used to identify the macrophages. CD68 and CD11b, and the major histocompatibility complex type II (MHCII) and CD4 and CD8 were analyzed. The RNAscope technique was used for fractalkine gene localization.

**Results:** Many macrophages were found around blood vessels in the stria vascularis but not CD4 and CD8 lymphocytes. Amoeboid macrophages were identified in the spiral ganglion with surveilling “antennae” projecting against targeted cells. Synapse-like contacts were seen on spiral ganglion cell bodies richly expressing single *CXC3CL* gene transcripts. Branching neurite-like processes extended along central and peripheral axons. Active macrophages were occasionally found near degenerating hair cells. Some macrophage-interacting T lymphocytes were observed between the scala tympani wall and Rosenthal's canal. CD4 and CD8 cells were not found in the organ of Corti.

**Conclusions:** The results indicate that the human cochlea is equipped with macrophages and potentially lymphocytes, suggesting both an innate and adaptive immune capacity. A rich expression of fractalkine gene transcripts in spiral ganglion neurons suggest an essential role for auditory nerve protection, as has been demonstrated experimentally. The findings provide further information on the important role of the immune machinery present in the human inner ear and its potential to carry adverse immune reactions, including cytotoxic and foreign body responses. The results can be used to form a rationale for therapies aiming to modulate these immune activities.

## Introduction

Acoustic over-stimulation may lead to cochlear inflammation activating cells of the monocyte-macrophage lineage to dispose of cells and waste material within the organ of Corti (OC) (1–5). Resident phagocytic cells are activated within the cochlea but can also be recruited from the bone marrow after aminoglycoside ototoxicity (6, 7) and selective hair cell damage (8). Macrophages (CD163, IBA1, and CD68) are also present in the human cochlea, even in cases with no history of hearing or balance disorders (9). Super-resolution structured illumination microscopy (SR-SIM) performed in our laboratory also displayed macrophages in the human cochlea and acoustic nerve, strongly expressing the marker IBA1 (ionized calcium-binding adaptor molecule 1) and CD11b (10, 11). The cells belong to the innate immune system but can ignite adaptive immune responses. Macrophages have the unique ability to switch from a healing (M2) to a killing mode (M1), a plasticity acting for organism survival (12, 13). Macrophages play a Janus-like double-faced role that can transition from a protective anti-inflammatory state to injurious pro-inflammatory phenotypes by intricate biochemical and cytokine/chemokine signaling systems (14, 15). The function of these cells may shed new light on inherent protective/destructive immune mechanisms residing in the human inner ear and how they relate to diseases such as secretive autoimmune and neuro-inflammatory conditions for which better diagnostic tools and treatments are warranted. They may evolve severe inflammatory reactions caused by bacterial and viral infections, causing collateral damage. Results show that the human cochlea possesses the cellular armamentarium necessary to immunologically surveil, heal, and protect the inner ear. In this review, we examined human cochlear macrophages and immune cells and localized for the first time chemokine/*CX3CL1* gene expression using RNAscope^®^ *in situ* hybridization. The technique detected target RNA in formaldehyde-fixed sections using probes amplifying target signals with no background noise from non-specific hybridization. Fractalkine signaling has been found to enhance survival of spiral ganglion neurons after hair cell damage by enrollment of macrophages (16) and also influences synaptic and neuronal recovery after noise injury (17). Our study's results show the rich expression of *CX3CL1* single molecule gene transcripts in the human spiral ganglion. Furthermore, alleged dendritic cells are believed to be involved in the restoration of the human reticular lamina following hair cell loss.

## Materials and Methods

The technique that we used for sampling human tissue was previously described (18). Specimens were from patients suffering from life-threatening posterior cranial fossa meningioma compressing the brain stem (19). The surgeons used a trans-cochlear approach to reach the clivus region. The operations were performed at Uppsala University Hospital by the Oto-neuro-surgical team. Seven cochleae were removed.

### Ethic Statements

The study of human materials was approved by the local ethics committee (no. 99398, 22/9 1999, cont, 2003, no. C254/4; no. C45/7 2007, Dnr. 2013/190), and patient consent was obtained. The study adhered to the rules of the Declaration of Helsinki. Archival sections from adult cochleae were used.

### Immunohistochemistry of the Human Cochleae

Immunohistochemistry procedures on human cochlear sections were described in previous publications (20). In short, the tissue was fixed in a solution of 4% paraformaldehyde phosphate buffer solution. The cochleae were decalcified in 10% ethylene-diamine-tetra-acetic acid (Na-EDTA) solution at pH 7.2 for 4 weeks. They were embedded in Tissue-Tek OCT embedding compound (Polysciences, Inc., Warrington, PA, USA), frozen, and sectioned at 8–10 μm using a cryostat microtome. Sections were incubated with an antibody solution under a humidified atmosphere at 4°C for 20 h. Sections were incubated with secondary antibodies conjugated to Alexa Fluor (Thermo Fisher Scientific, Uppsala, Sweden) counterstained with the nuclear stain 4',6-diamidino-2-phenylindole dihydro-chloride (DAPI), mounted with ProLong^®^ Gold Antifade Mountant (Catalog number: P10144; Thermo Fisher Scientific, Uppsala, Sweden), and then covered with cover glass compatible with confocal and super-resolution microscopes. Primary and secondary antibody controls and labeling controls were used to exclude endogenous reaction products (21).

### Antibodies

Table 1 shows the antibodies used in this study. The antibody against type IV collagen was used to define the basal lamina of neurons, blood vessels, and epithelium. We used a polyclonal antibody (1:25, goat ab, AB769; Millipore AB, Sweden). For resident macrophages, we used the antibody against IBA1 (polyclonal, 1:100, rabbit, PA527436 from Invitrogen, Sweden). Specificity was proven by IBA1 antibody blotting (22). The fractalkine antibody was a monoclonal antibody (1:100, mouse, MAB3651; R&D Systems, Minnesota, USA). This antibody's specificity was verified in a western blotting experiment (23). Information about the primary and secondary antibodies is shown in Table 1. Patient age ranged from 40 to 70 years. Four patients had normal audiograms and two had 50 dB loss at ranges 1–8 kHz and 2–4 kHz, respectively. Two cochleae were reanalyzed using TEM and LM.

**Table 1:** Antibody table.

**Antibody**	**Type**	**Host**	**Species reactivity**	**Dilution**	**Catalog number**	**Producer**
IBA1	P	Rabbit	H, M, R	1:100	PA527436	Invitrogen
CX3CL1	M	Mouse	H	1:100	MAB3651	RandD Systems
CX3CR1	P	Rabbit	H	1:50	PA5-19935	Invitrogen
P2Y12	P	Rabbit	H	1:50	PA5-34079	Invitrogen
MHC II	M	Mouse	H	1:50	MA5-11966	Invitrogen
CD11b	M	Rabbit	H	1:50	AB52478	Abcam
CD117	M	Rat	H, M, R	1:100	14-1172-82	Invitrogen
CD19	M	Rat	H, M, R	1:100	14-0194-82	Thermo Fisher Scientific
CD8α	M	Mouse	H	1:100	MAB1509	RandD Systems
CD4	P	Goat	H	1:150	AF-379-NA	RandD Systems
CD68	M	Mouse	H, M, R	1:50	NB100-683	Novus
TLR4	OC	Rabbit	H, M	1:10	710185	Thermo Fisher Scientific
TMEM119	P	Rabbit	H	1:500	ab185333	Abcam
Vimentin	M	Mouse	H, M, R etc.	1:50	V5255	Sigma-Aldrich
Collagen IV	P	Goat	H, M, Bovine	1:10	AB769	Millipore

*Invitrogen, Waltham, USA. R and D Systems, Minneapolis, USA. Thermo Fisher Scientific, Waltham, USA. Sigma-Aldrich, Saint Louis, MO, USA. Abcam, Cambridge, UK. Novus, Littleton, USA. Millipore, Burlington, USA. P, Polyclonal; M, Monoclonal; OC, Oligoclonal; H, Human; M, Mouse; R, Rat*.

### Imaging and Photography

Sections for immunohistochemistry were first investigated with an inverted fluorescence microscope (Nikon TE2000) equipped with a spot digital camera with three filters (for emission spectra maxima at 358, 461, and 555 nm). Image-processing software (NIS Element BR-3.2, Nikon), including image merging and a fluorescence intensity analyzer, was installed on a computer system connected to the microscope. For laser confocal microscopy, we used the same microscope equipped with a three-channel laser emission system. For optical scanning and image processing, Nikon EZ-C1 (ver. 3.80) software was used. SR-SIM was performed using a Zeiss Elyra S.1 SIM system and a 63×/1.4 oil Plan-Apochromat 63×/1.4 oil objective (Zeiss), sCMOS camera (PCO Edge), and ZEN 2012 software (Zeiss). Multicolor SR-SIM imaging was attained with a laser and filter setup: first channel−405 nm laser excitation and BP 420–480 + LP 750 filter; second channel−488 nm laser excitation and BP 495–550 + LP750 filter; and third channel−561 nm laser excitation and BP 570–620 + LP 750 filter. To maximize the image quality, five grid rotations and five phases were used for each image plane and channel. The grid size was adjusted by ZEN software for each wavelength of excitation. SR-SIM images were processed with ZEN software and theoretical point spread function (PSF) calculations. The microscope is capable of achieving a lateral (*X*–*Y*) resolution of ≈100 nm and an axial (*Z*) resolution of ≈300 nm (24). The resolution of the SIM system using the BioVis platform at Uppsala University was measured with sub-resolution fluorescent beads (40 nm, Zeiss) in the green channel (BP 495-550 + LP750).

### Field Emission Scanning Electron Microscopy (FE-SEM)

Two human cochleae removed earlier were re-evaluated (25). Opened windows ensured fast penetration of the fixative. Inner ears were fixed with ice-cold Karnowsky's formaldehyde–glutaraldehyde solution (4% paraformaldehyde with 0.1% glutaraldehyde in 0.1 M cacodylate buffer), post-fixed in 1% osmium-tetra-oxide in 0.05 M cacodylate buffer for 1 h, washed in phosphate-buffered saline (PBS), pH 7.4, dehydrated in graded ethanol (70, 80, 90, 95, and 100% at 10 min each), critical point dried and attached to aluminum stubs. The specimens were coated in a BALTECH MED020 Coating System with gold–palladium to a nominal depth of 10–12 nm and viewed in a ZEISS DSM982 Gemini Field Emission Electron Microscope operating at 5 kV. Maximal resolution at this voltage was estimated to be approximately 2 nm. Digital photos were taken at 1280 × 1024 ppi resolution in TIFF format and stored on a zip-drive. Measurements were performed using Image Pro 4.5.1.29 (Media Cybernetics, Inc., Rockville, MD, USA) image analysis software. The specimens were analyzed with special reference to free cells and macrophage activity.

### Transmission Electron Microscopy (TEM)

Archival sections were analyzed from specimens collected during surgery and earlier publication. The specimens were fixed in 3% phosphate-buffered glutaraldehyde, pH 7.4, and rinsed in 0.1 M cacodylate buffer. Thereafter, they were placed in 0.1 mol/L Na-EDTA for 4 weeks at room temperature. Decalcification was checked by radiography. The tissue was rinsed and stained with 1% sodium phosphate-buffered osmium tetroxide for 1 h and then dehydrated in graded ethanol and embedded in epoxy resin in a vacuum chamber for 4 h. Ultrathin sections were cut with a Leica UltracutS microtome and transferred to pioloformF (polyvinylacetate) coated (1.5% pioloform in chloroform) slot grids. Staining was performed with an automated system (Leica EM Stain) with uranyl acetate (5 g/l; 30 min) and lead citrate (5 g/l, 50 min) at 25°C, and sections were analyzed on a Zeiss EM109 transmission electron microscope.

### RNAscope^®^ Protocol

Human cochlear tissue was fixed with freshly prepared 4% paraformaldehyde in 1x PBS according to Bio-Techne's recommendations (24 h, 4°C). Sections were obtained in the same way as described in the immunohistochemistry method and stored in a −70°C freezer. RNA *in situ* hybridization (ISH) was performed using the RNA-scope^®^. The RNA-scope^®^ Reagent Kit (Bio-Techne, Minneapolis, MN, USA) was used according to the manufacturer's instructions (kit version 2). Briefly, sections underwent air drying in a −20°C freezer (moved from a −70°C freezer) for 1–2 h. Then, the sections were washed in 1x PBS buffer solution for 10 min, removing the OCT embedding matrix compound. The sections were baked at around 60°C for 30 min to increase the adhesion of the sections to the slide. Post-fixation of the sections was carried out using freshly prepared 4% paraformaldehyde in 1x PBS at 4°C for 15 min. RNA-scope^®^ hydrogen peroxide solution was applied to the sections (10 min, RT) and washed with distilled water, followed by pre-treatment target retrieval and protease III incubation. The sections were bathed in the RNA-scope^®^ 1x target retrieval solution in a steamer (80°C, 5 min) then incubated in RNA-scope^®^ protease III solution (30 min, 40°C). After protease III incubation, the sections were washed with distilled water or 1x PBS before being subjected to an RNA-scope^®^ hybridization assay. The paired double-Z oligonucleotide probes were designed and produced by Bio-Techne and depended on the targets' FASTA format nucleotide sequences provided on the NCBI database. Table 2 provides information about the probes used in the present study. To start the hybridization, the RNA probe fluid was warmed in a 40°C water bath for 10 min, and then cooled down to room temperature. The probe was diluted with probe diluent (Cat. No. 300041, 1:50). The probe was added to the sections and incubation was carried out in a HybEZ™ Oven (Bio-Techne Co.) for 2 h at 40°C. After hybridization incubation, the slides were washed using 1x RNA-scope wash buffer. Then, sections were incubated with RNA-scope Multiplex FL v2 Amp 1, Amp 2, and Amp 3 (for 30/30/15 min, respectively) sequentially at 40°C to amplify the signal. For signal development, RNAscope^®^ Multiplex FL v2 HRP-C1 (for CX3CL1) and HRP-C3 (for GJB6) were added to the sections (incubation time 15 min, 40°C) in our RNA-scope^®^ Multiplex study. For revealing signals, TSA-diluted Opal™ 570 fluorophore were added to sections after HRP-C1 and C3 (washed away HRP C1 and C3 using 1x RNA-scope^®^ wash buffer), incubating sections for 30 min at 40°C. In the present study, channel one was assigned to the CX3CL1 probe (C1) and channel three to the GJB6 probe (C3). After fluorophore incubation and rinse with 1x RNAscope^®^ wash buffer, RNA-scope^®^ Multiplex FL v2 HRP blocker was added and incubated in the oven at 40°C for 15 min. Finally, the sections were counterstained with RNAscope^®^ DAPI (30 sec), and the slides were cover-slipped with ProLong^®^ Glass Antifade Mountant (Thermo Fisher Scientific). RNA-scope ISH produces a punctum-like signal, and each represents a single mRNA transcript (26). The dapB probe was used as a negative control. DapB is only present in a very rare strain of soil bacteria. Therefore, the probe should not reveal signals in human tissue. Our RNA-scope control result was consistent with a feasible RNAscope^®^ technical protocol, hence supporting the specificity of the *CX3CL1* and *GJB6* signals we observed in the human cochlea.

**Table 2:** Gene Information for RNAscope probe designing or purchase.

**Gene name**	**Species**	**Gene ID**	**Chromosome location**	**Probe**	**Producer**
CX3CL1	Human	6376	16q21	411261	BioTechne
GJB6	Human	10804	13q12.11	541391	BioTechne
CX3CR1	Human	1524	3p22.2	411251	BioTechne
AIF1	Human	199	6p21.33	433121	BioTechne

*BioTechne, R and D Systems, Minneapolis, USA. Allograft inflammatory factor 1 protein is known as ionized calcium-binding adapter molecule 1 (IBA1) and is encoded by the AIF1 gene in human*.

## Results

### IBA1 Cells in the Human Cochlea

**Figure 1:**
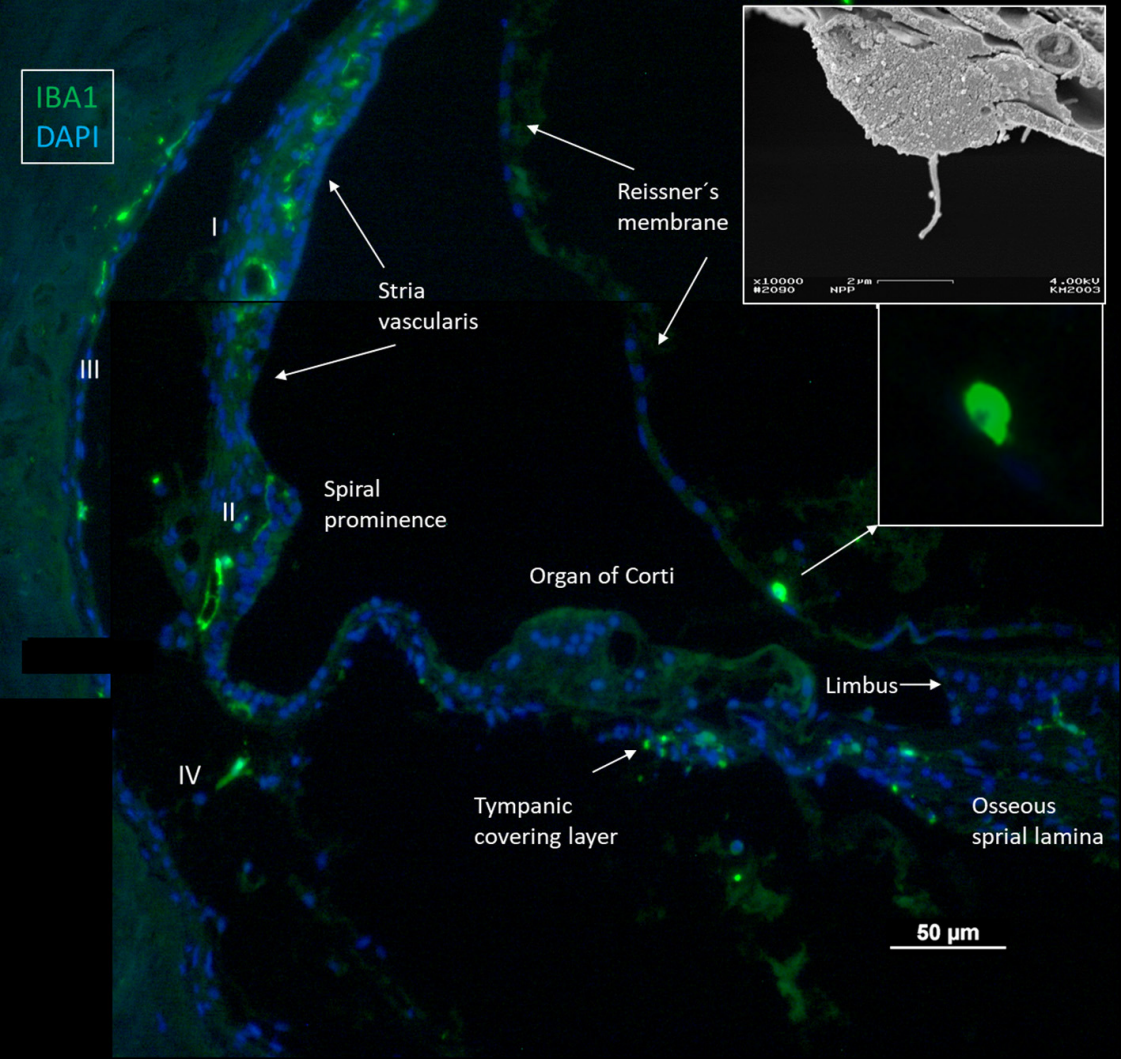
Immunofluorescence of a radial section of a human cochlear duct showing the distribution of IBA1 cells. In the spiral prominence, some IBA1 cells almost reach the endolymph. Scanning electron microscopy shows a free cell transitioning a cell border in the human cochlea (upper inset). Reissner's membrane (RM) may also contain macrophages (lower inset). In the SV, IBA1 cells are mostly found in the middle layer close to the blood vessels. No IBA1 cells can be observed in the organ of Corti in this section. I–IV; fibrocytes.

Confocal microscopy and SR-SIM revealed a multitude of IBA1 positive macrophages in the human cochlea (Figure 1), including the lateral cochlear wall, tympanic covering layer (TCL), spiral limbus, Reissner's membrane (RM), and osseous spiral lamina (OSL). The wall of the scala vestibuli and tympani also contained IBA1 cells. Perivascular macrophages were also present in the scala tympani. Macrophages expressed the fractalkine receptor CX3CR1 (Supplementary Figure 1).

### Spiral Ligament and Stria Vascularis (SV)

**Figure 2:**
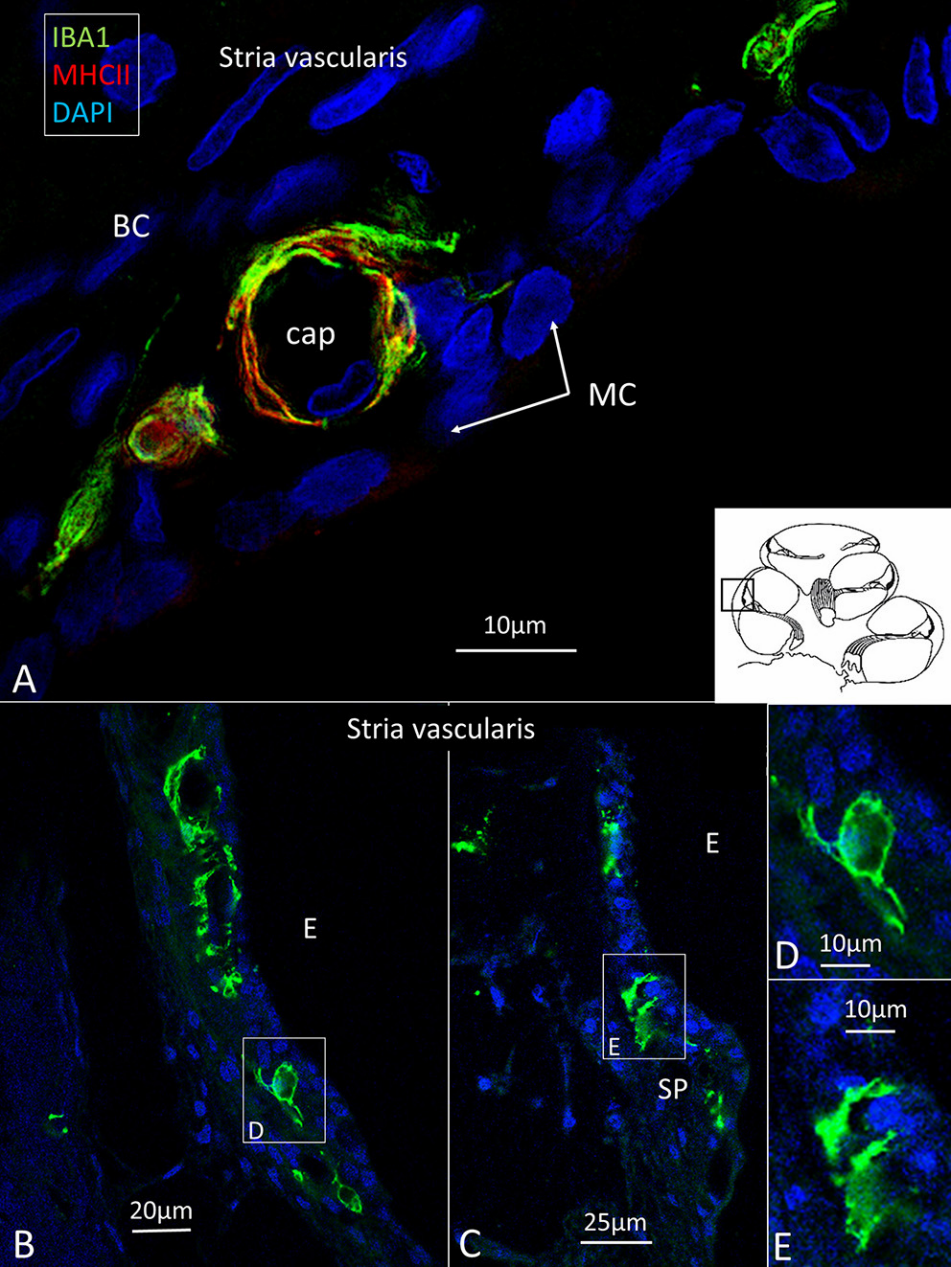
**(A)** IBA1 cells located in the intermediate layer of the SV surrounding blood vessels. The cells also express MHC II. BC: basal cell. MC: marginal cell. **(B–D)** Macrophages located in the SV and spiral prominence. **(E)** Some cells reach areas between epithelial cells.

IBA1 cells frequently expressed the major histocompatibility complex type II (MHC II), and in the SV cells were mostly located in the middle cell layer surrounding blood vessels (Figure 2). Cells could be multi-branched, rounded, or elongated, and nuclear chromatin typically contained IBA1 protein. Some cells had long pseudopodia with terminal knobs reaching the wall of the capillaries. In some cases, the lumen of the vessels contained IBA1 cells, assumingly representing blood monocytes. The IBA1 cells did not contain melanin granules. The cells did not express TMEM119, a microglia marker believed to be exclusive on a subset of IBA1, CD68 microglia with ramified and amoeboid morphologies in the brain (27). Slender IBA1 cells sometimes reached between epithelial cells in spiral prominence (SP). Macrophages were also seen in the spiral ligament among type II, IV, and V fibrocytes but rarely among type I fibrocytes. A few type III fibrocytes and/or cells facing the endosteum also seemed to express IBA1 (Figure 1). If these cells expressed CD45 and MHCII could not be assessed with certainty.

### Organ of Corti

**Figure 3:**
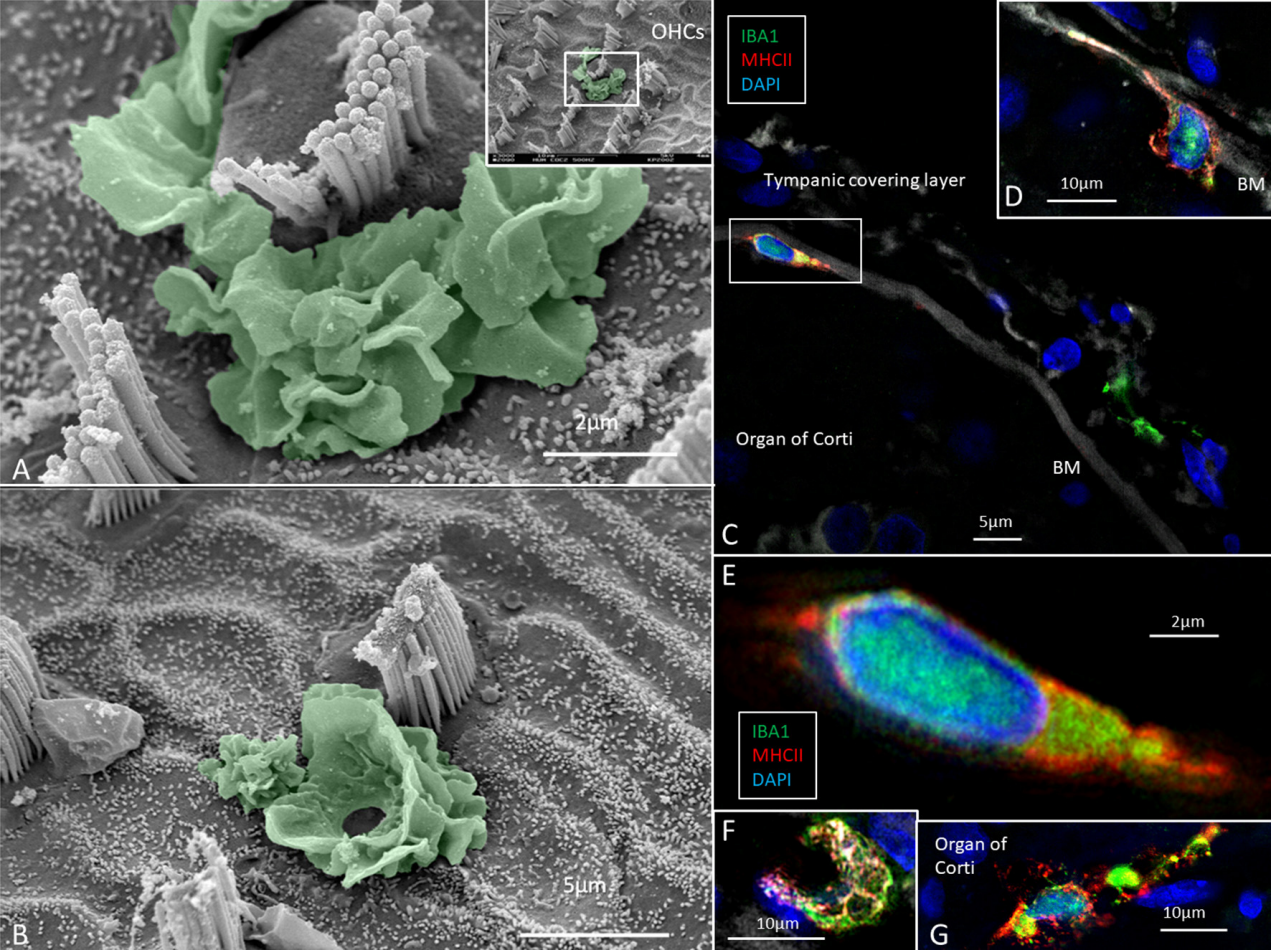
**(A,B)** SEM of the human organ of Corti (OC) shows alleged dendritic cells surrounding two outer hair cells (OHCs). The cell in B encloses a hole in the reticular lamina (*), probably from a missing OHC. **(C,D)** SR-SIM shows IBA1 positive macrophages on the basilar membrane facing the OC. Framed area is shown in higher magnification in **(E)**. The cell express IBA1 and MHC II. **(F)** A macrophage on the scala tympani side of the basilar membrane also expresses MHC II. **(G)** Macrophages located on the basilar membrane in the OC.

Several IBA1 cells were seen among the peripheral axons at the habenula perforata (HP) and a few occasionally in the organ of Corti (OC), even associated with hair cells (Figures 3A,B). Re-evaluating archival scanning electron microscopy (SEM) specimens demonstrated occasionally alleged dendritic macrophages adjoining outer hair cells (OHCs). A characteristic feature revealed by SEM was their large cytoplasmic veils or folded cell coat. Their folded cell coat enclosed the apical portion of the OHC, suggesting a closure of the reticular lamina in order to “heal” it after the loss of the sensory cell (Figure 3B). SIM also demonstrated IBA1 cells in the organ of Corti expressing MHC II (Figures 3C–G). MHC II was mostly membrane-associated but could occasionally appear in cytoplasmic masses.

### IBA1 Cells in the Spiral Ganglion and Tympanic Covering Layer (TCL)

**Figure 4:**
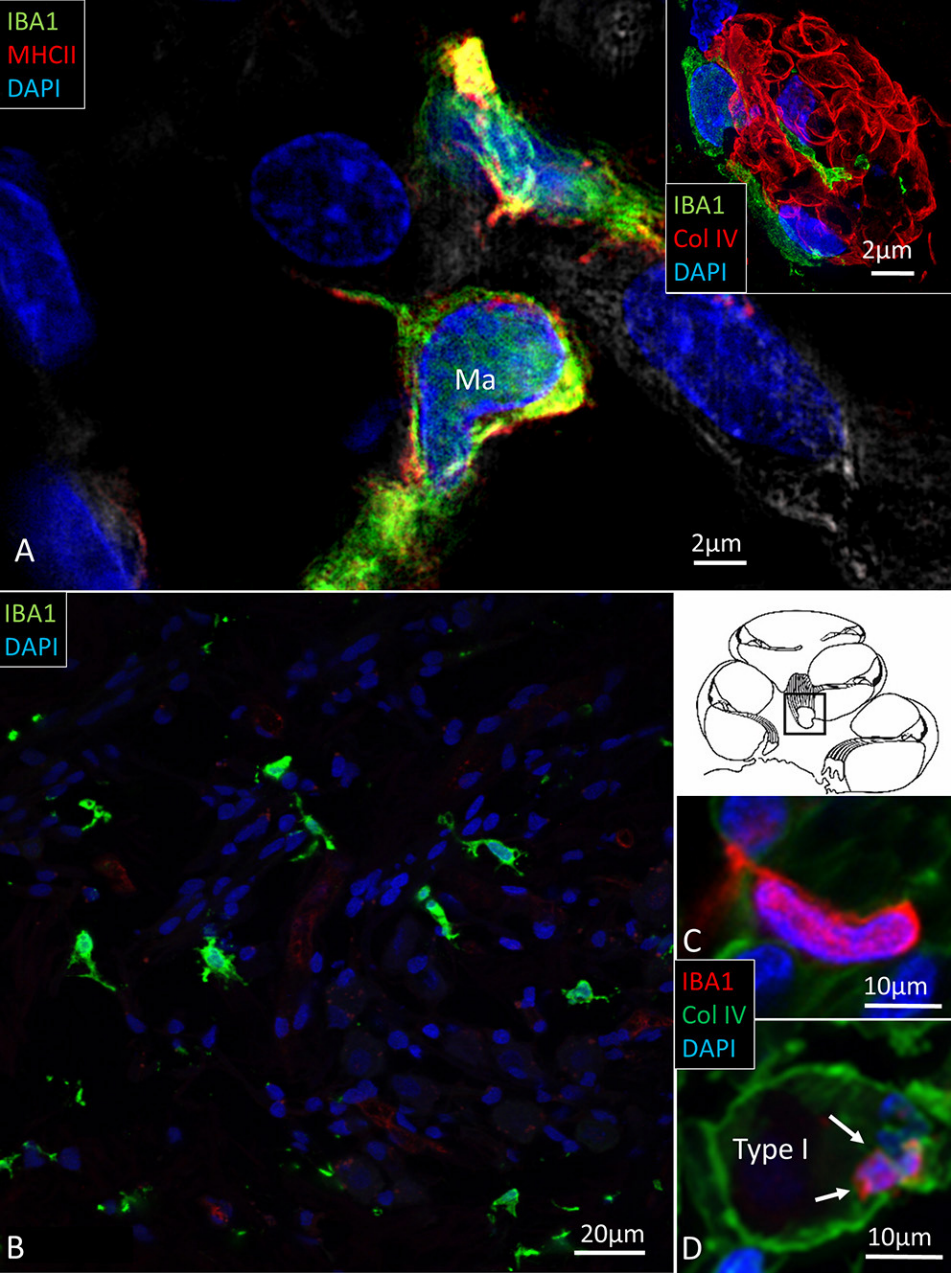
**(A)** SR-SIM of IBA1 cells co-expressing MHCII in the human spiral ganglion. IBA1 cells mingle with Schwann cells in a peripheral axonal bundle (inset). **(B)** Confocal microscopy of several branched IBA1 macrophages is seen in the spiral ganglion. **(C)** The macrophages have nuclei expressing IBA1 protein. **(D)** The basal lamina surrounding the satellite cells expresses collagen IV. A macrophage (arrows) is closely related to the satellite glia cell surrounding a type I ganglion cell. The IBA1 cell is seen to cross the basal lamina.

**Figure 5:**
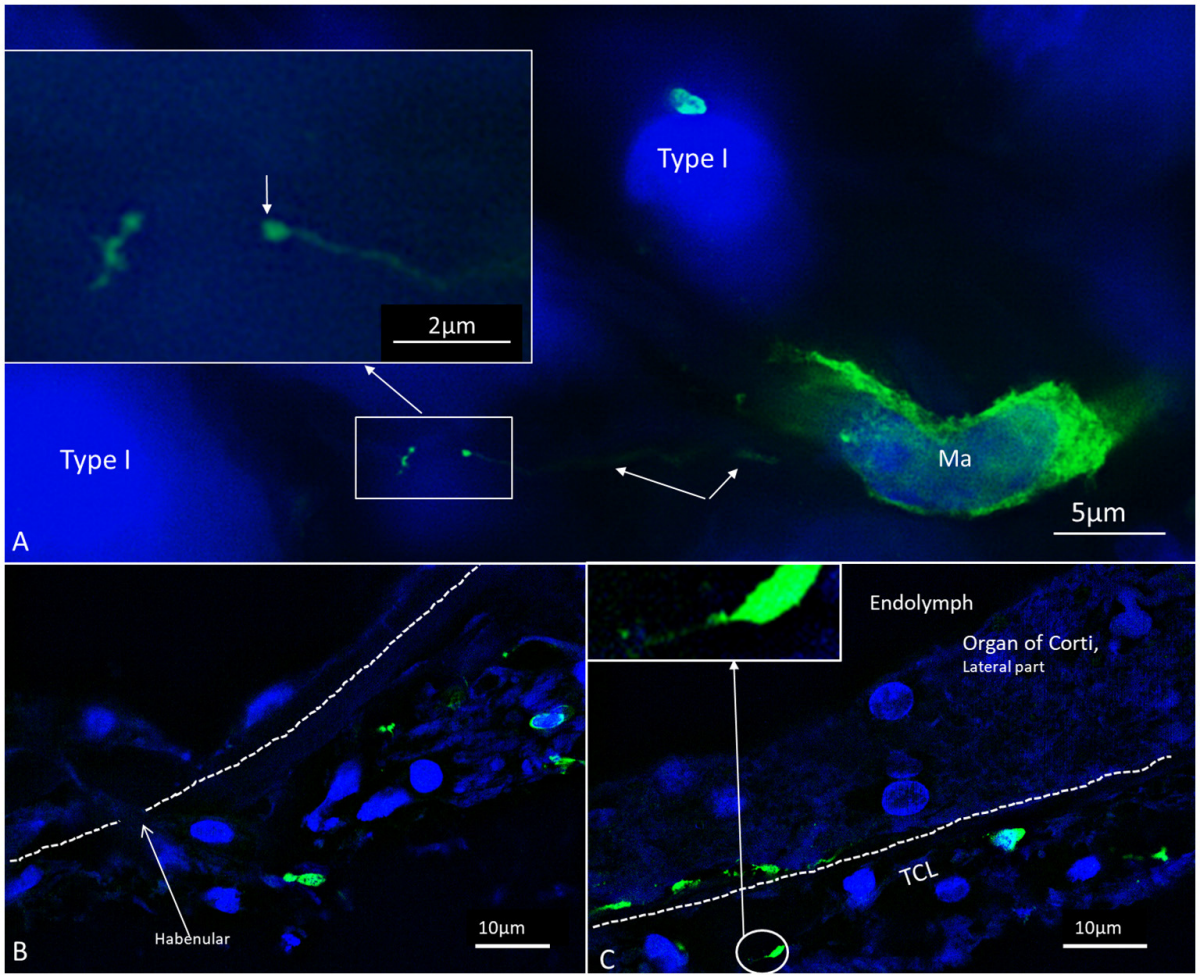
SR-SIM of macrophages in the spiral ganglion **(A)** and OC **(B,C)**. The cells exhibit thin (0.1–0.2 μm) filamentous processes extending into the extracellular tissue to adhere to surrounding cells ending with a small knob (arrow). The dimension suggests that cell processes may reach the OC for surveillance through the HP.

**Figure 6:**
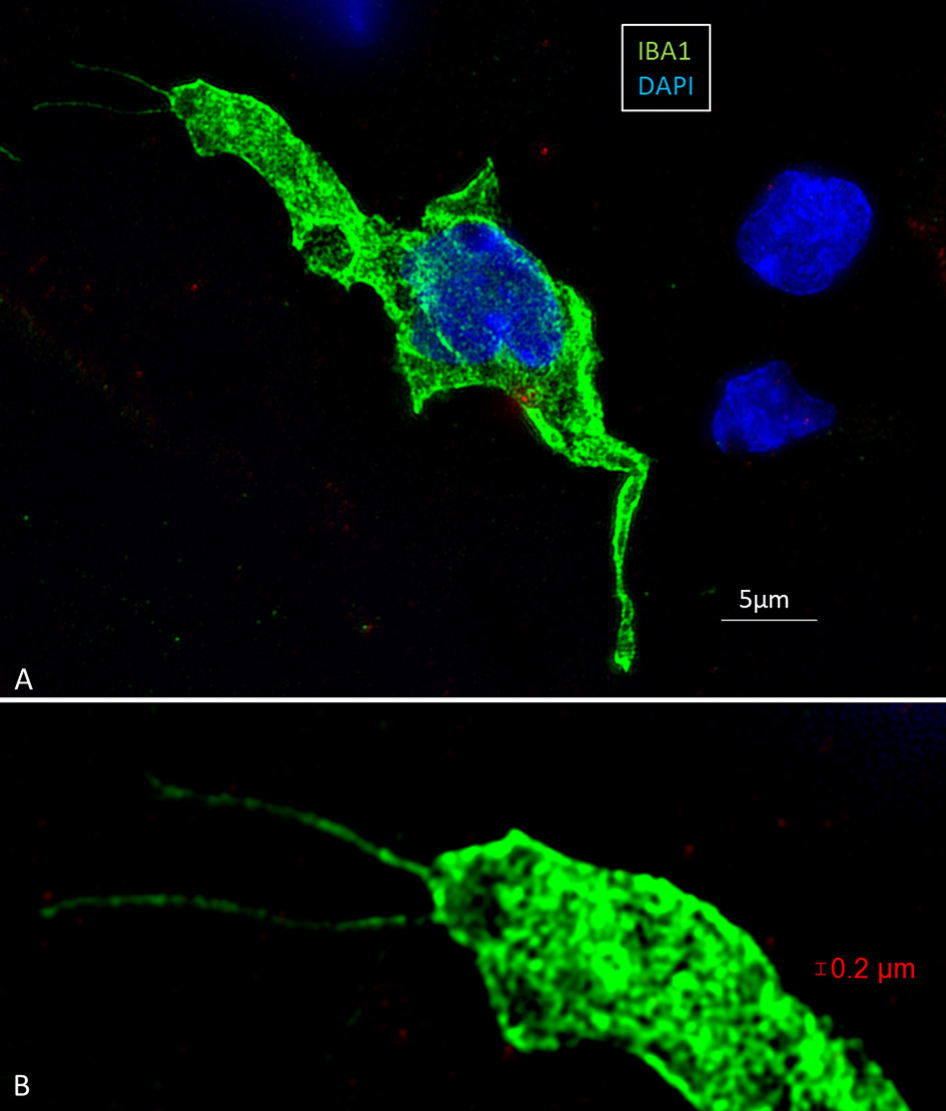
**(A)** SR-SIM of an amoeboid human macrophage located in the human cochlea. **(B)** The cell is endowed with long processes or “antennae” which are apparently involved in the scanning of the microenvironment. The IBA1 protein (green) fills the entire cells and is also typically located in the nucleoplasm (DAPI). Note the presence of large intracytoplasmic vesicles. From Frontiers in Immunology 2018 (10).

**Figure 7:**
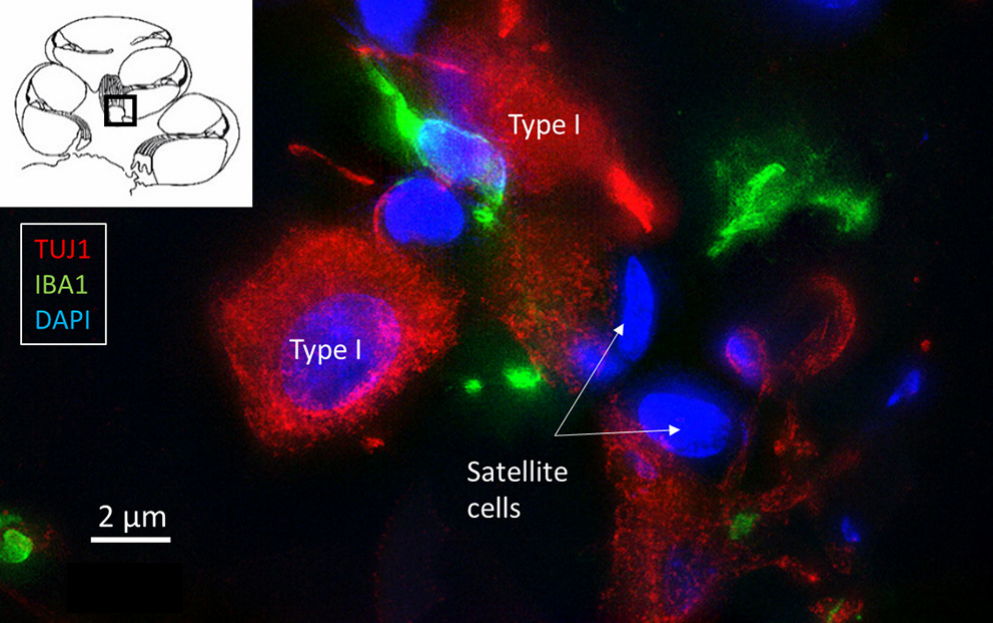
SR-SIM of IBA1 cells (green) contacting type I spiral ganglion cells (red).

Multiple IBA1 cells were found in the Rosenthal canal (RC) around the spiral ganglion and satellite glial cells. They also expressed MHCII (Figure 4). Some cells were positive for CD11b, a marker of macrophages and microglia, as well as CD68 and CD45 (not shown). Cells expressing IBA1 were branched and slender with highly variable anatomy. Using high-resolution microscopy, it was possible to identify remarkable amoeboid specializations and variants around the spiral ganglion cell bodies. The macrophages were endowed with thin (0.2 μm) “antennae-like” processes projecting into the extracellular tissue (Figures 5, 6). Synapse-like processes were directed against TUJ1-positive nerve soma. Macrophages physically interacted with the spiral ganglion cells and adhered to the basal lamina of the satellite cells, particularly at the central and peripheral axonal initial segments (AIS) (Figure 7). At some places, the IBA1 cells perforated the basal lamina and reached the space between the satellite cells and nerve soma. Some cells in the TCL were IBA1 positive and also showed long antennae-like processes (Figures 5B,C). These cells seemed firmly attached to the undersurface of the basilar membrane (BM).

### Macrophages in Central and Peripheral Axons

**Figure 8:**
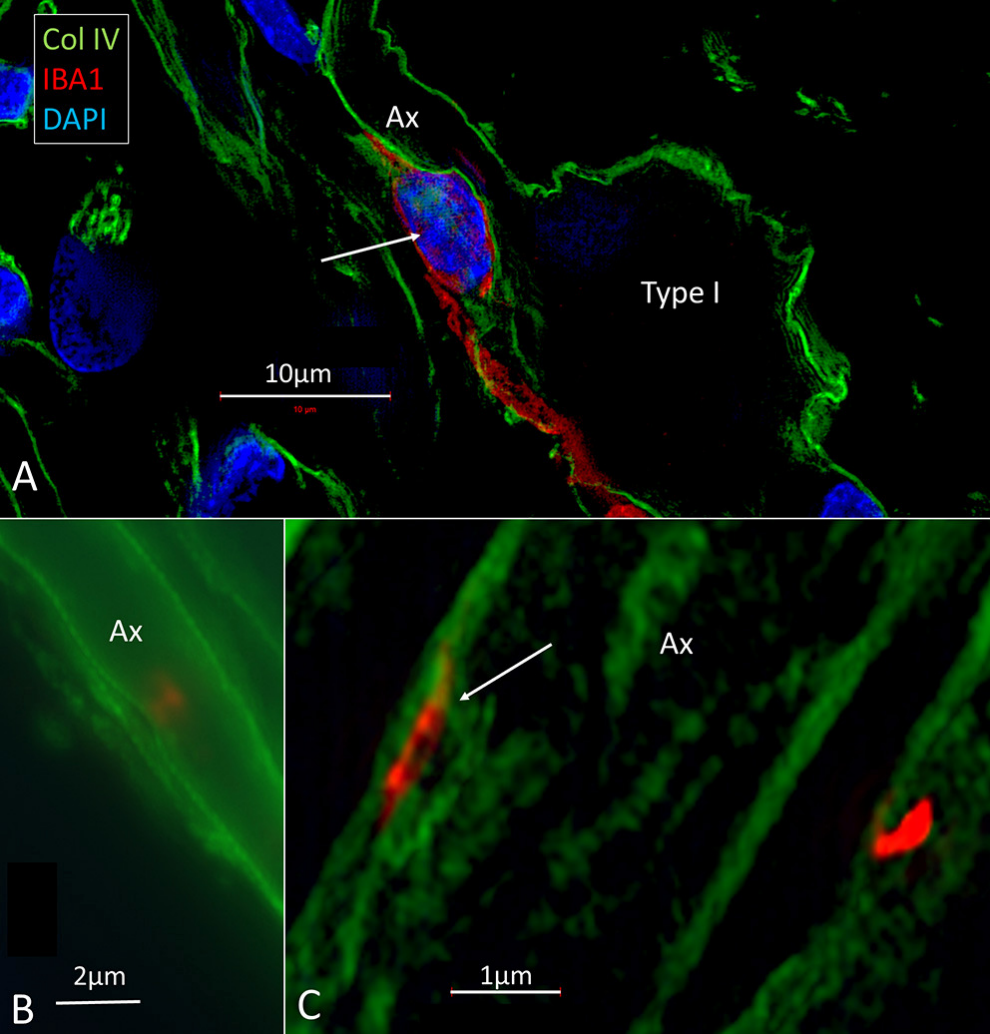
**(A)** A macrophage (red)can be seen lying close to the spiral ganglion cell body. The macrophage cell nucleus also expresses IBA1. **(B,C)** Its cell processes extend along the axon and form contact points at the perineural spaces.

There was also a surprisingly large number of macrophages located among central and peripheral axons (Figure 8). They ran parallel to the central axons, with long and slender processes measuring up to 50 μm and having a diameter of ~0.5 μm. Their cell nuclei typically expressed IBA1. The processes had terminal swellings, and collagen IV and IBA1 co-staining demonstrated that these pseudopodia could extend across the basal lamina of the Schwann and enter the perineural space. One IBA1 cell could send several processes to adjacent nerve fibers within the osseous spiral lamina. The branches also penetrated into the axon, suggesting that the macrophage process lay inside the myelin layer (Figures 8B,C). Serial photos and 3D reconstructions verified these conditions. If the IBA1 branches adhered directly to the axonal cell membrane or were positioned at the Ranvier nodes, could not be assessed with certainty. IBA1 cells also surrounded the unmyelinated nerve fibers beneath the HP, but it could not be verified if macrophages entered the HP and reached into the OC (Figures 5B,C).

### Expression of CX3CL1 in the Human Cochlea

**Figure 9:**
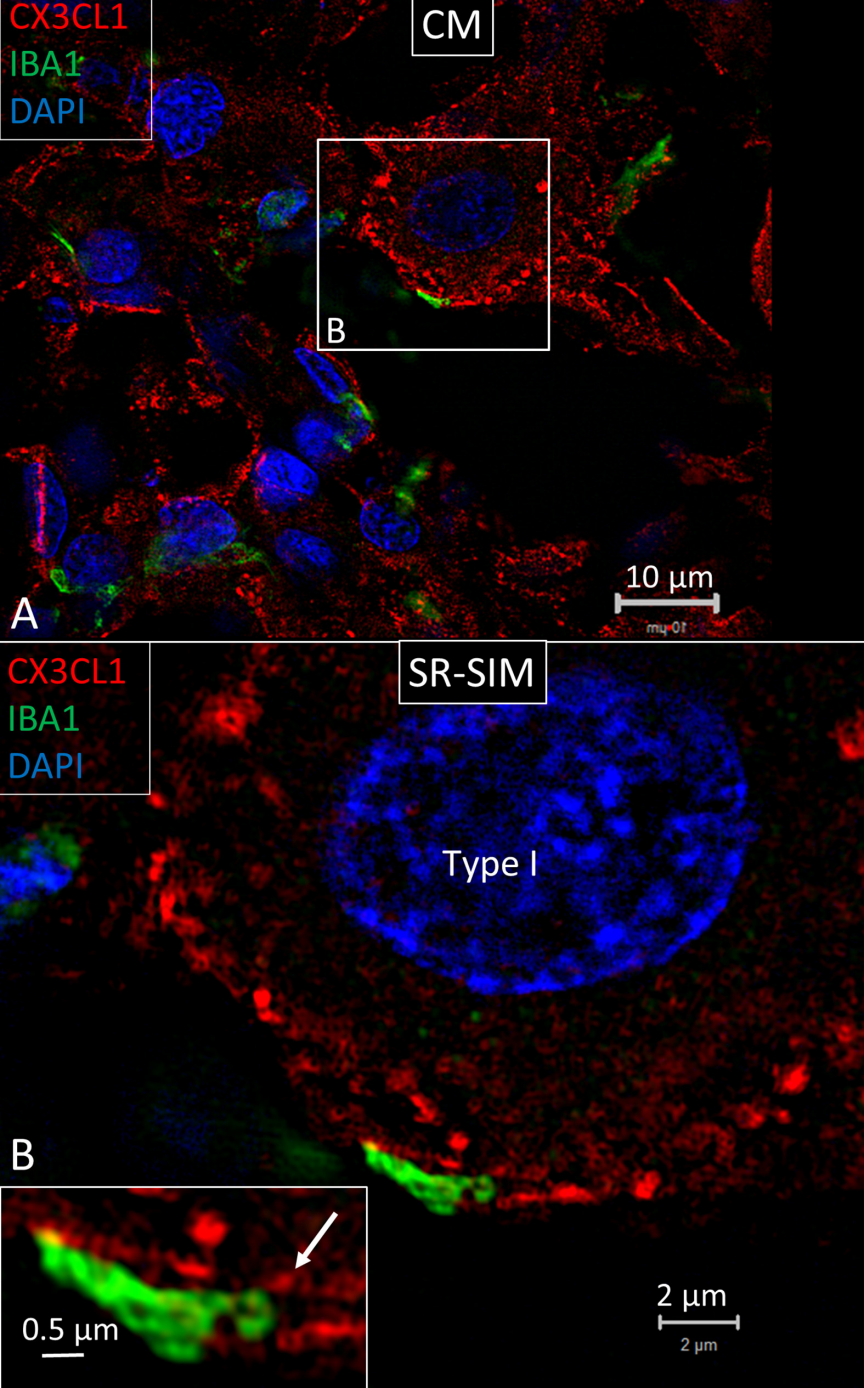
**(A)** SR-SIM showing expression of fractalkine in the human spiral ganglion. Framed area is magnified in **(B)**. **(B)** An IBA1 cell process adheres to the cell coat of a type I spiral ganglion cell. It contains a small cell process projecting into the nerve cell soma (arrow in inset).

Under confocal microscopy, spiral ganglion macrophages were diffusely stained with antibodies directed against CX3CL1 (Figure 9A). SR-SIM showed focal membrane expression of CX3CL1 protein on the spiral ganglion cell bodies (Figure 9B). IBA1 cell processes adhered to the cell coat and focal deposits and small cell processes could be seen projecting from the macrophage into the cell body (Figure 9B, inset). Cells within the OC, SV and spiral ligament showed little expression of fractalkine. No particular difference in staining was noted between hair cells and supporting cells. Cells of the TCL displayed some staining. Cochlear macrophages expressed CX3CR1 (Supplementary Figure 1).

### Expression of *CX3CL1* Gene Transcripts

**Figure 10:**
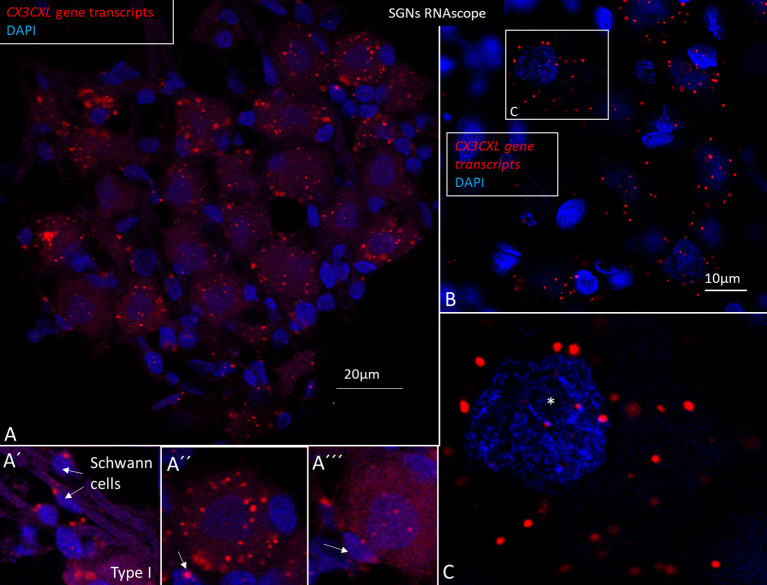
**(A–C)** SR-SIM and RNA-scope demonstrating *CX3CL1* gene transcripts in the human spiral ganglion. Each red punctum represents a single target molecule. Genes are mostly located in the cytoplasm of the type I ganglion cell bodies. Few puncta can also be observed in the axonal Schwann cells (A′) and surrounding satellite cells (A″, A^‴^). **(B)** Framed area of a type I spiral ganglion neuron is magnified in C. Both the cell nucleus and cytoplasm contain gene puncta. *nucleolus. SGN; spiral ganglion neuron.

SR-SIM displayed RNA single gene transcripts as red stained puncta. The *GJB6* gene encoding connexin30 (Cx30) served as an additional control since the protein localizes only in distinct cellular domains in the SV. Cx30 protein is expressed in the basal and intermediate cells but not in marginal cells (28). Positive and negative controls verified the specificity of gene localization. In RC, *CX3CL1* gene transcripts were mainly found in the type I spiral ganglion cell bodies (Figure 10). No nerve marker was used to identify the type I ganglion cells due to their characteristic nuclear DAPI staining. A few gene puncta were seen in surrounding cells such as satellite cells and Schwann cells in the myelinated axons. Mesenchymal cells in RC lacked gene puncta. RNAscope^®^ detection of *CX3CR1* (fractalkine receptor) and *AIF1 (*allograft inflammatory factor 1) genes were unsuccessful.

**Figure 11:**
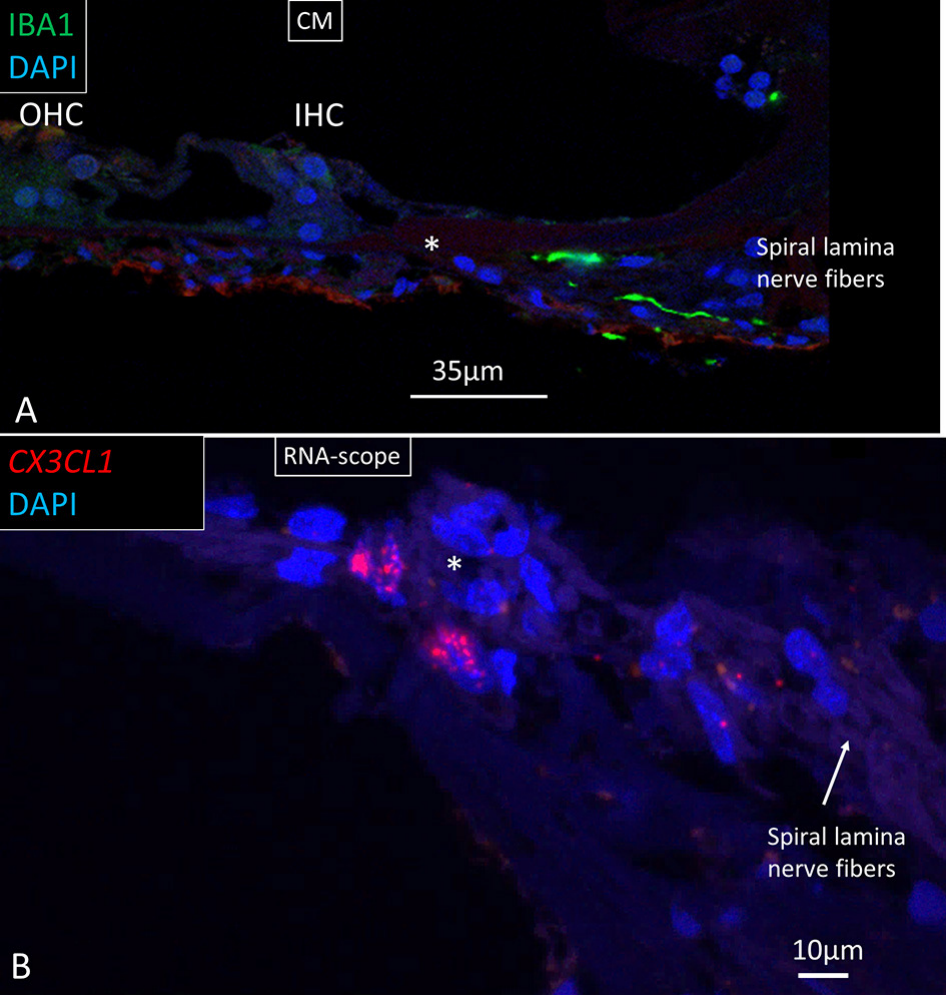
**(A)** Confocal image of IBA1 macrophages running along lamina nerve fibers. *HP. **(B)** SR-SIM and RNA-scope of fractalkine gene expression in cells surrounding the unmyelinated lamina fibers. IHC, inner hair cell; OHC, outer hair cell; CM; confocal microscopy.

**Figure 12:**
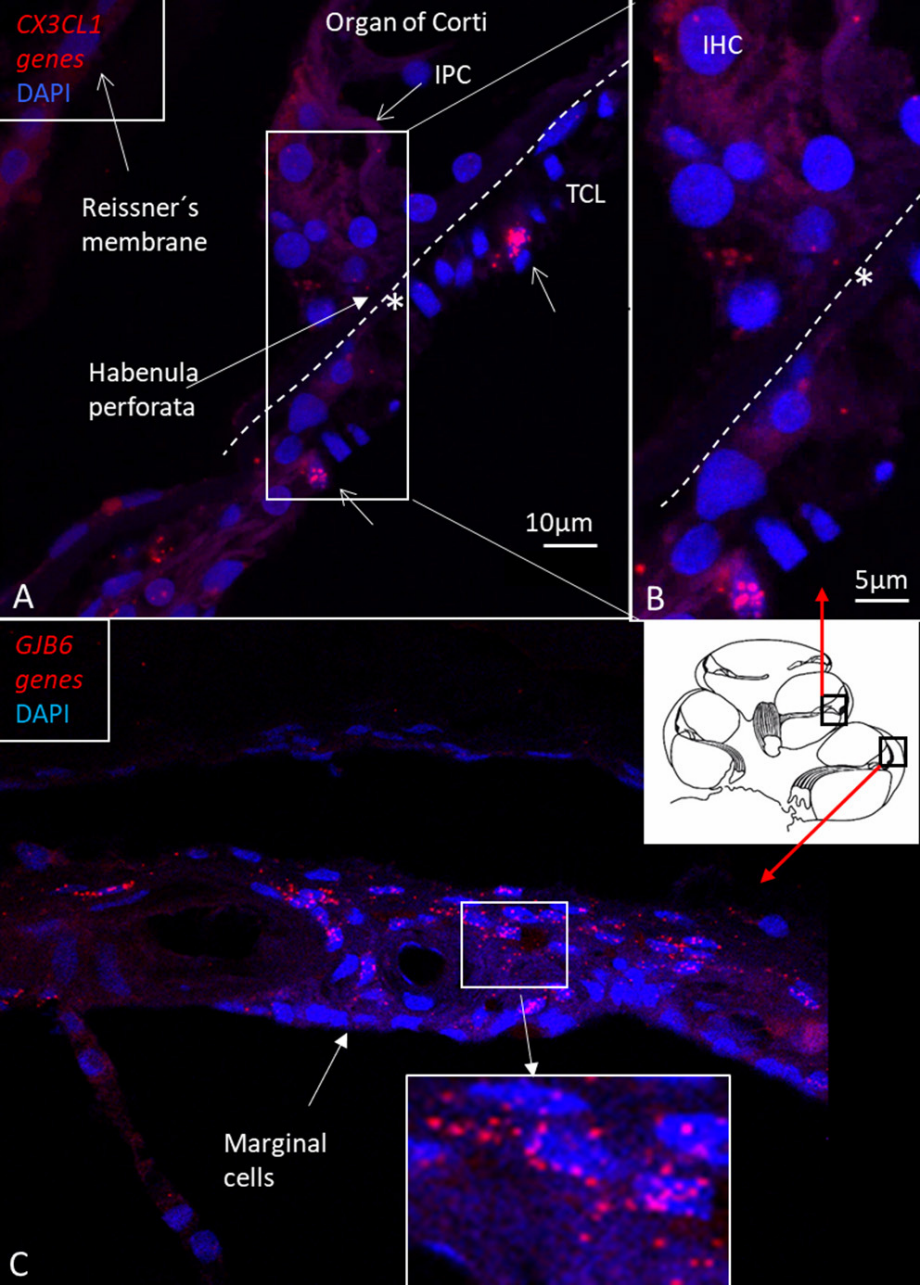
**(A)** RNA-scope ISH and SR-SIM of *CX3CL1* gene transcripts in the region of the IHC and BM (*). Framed area is magnified in **(B)**. Supporting cells contain a few puncta, mostly in cell nuclei. Some cells in the TCL contain numerous gene puncta (arrows). IPC: inner pillar cell. TCL: tympanic covering layer. IHC: inner hair cell. **(C)** RNA-scope ISH and SR-SIM of *GJB6* gene transcript localization in the lateral wall of the human cochlea. The framed area is magnified in the lower inset. Gene transcripts are restricted to cells located lateral to the marginal cells, which is consistent with Cx30 protein expression.

In the OSL, transcripts were restricted to the Schwann cells located in the nerve bundle's periphery beneath the HP. There were no *CX3CL1* genes expressed in the spiral lamina nerve fibers, but they were seen in cells surrounding the unmyelinated nerve bundles beneath the HP (Figure 11). Several IBA1 cells were localized at this location. There were few *CX3CL1* genes expressed in supporting cells of the OC (Figures 12A,B). A few puncta were seen in Hensen cell nuclei. Few genes were found in the SV and spiral ligament. *CX3CL1* gene transcripts were found in a few endothelial cells or cells surrounding the SV and SP vessels (Supplementary Figures 1A–C). The genes were mostly expressed in the cell nuclei. This contrasted to the large number of single GJB6 gene transcripts located in OC supporting cells, outer sulcus, and basal cells of the SV (Figure 12C). Several gene puncta were noted in some cell nuclei in the TCL. A few cells lining the endolymph space in the spiral lamina contained *CX3CL1* genes (Supplementary Figures 1D–F).

### CD4 and CD8 Cells in the Human Spiral Ganglion (SG)

**Figure 13:**
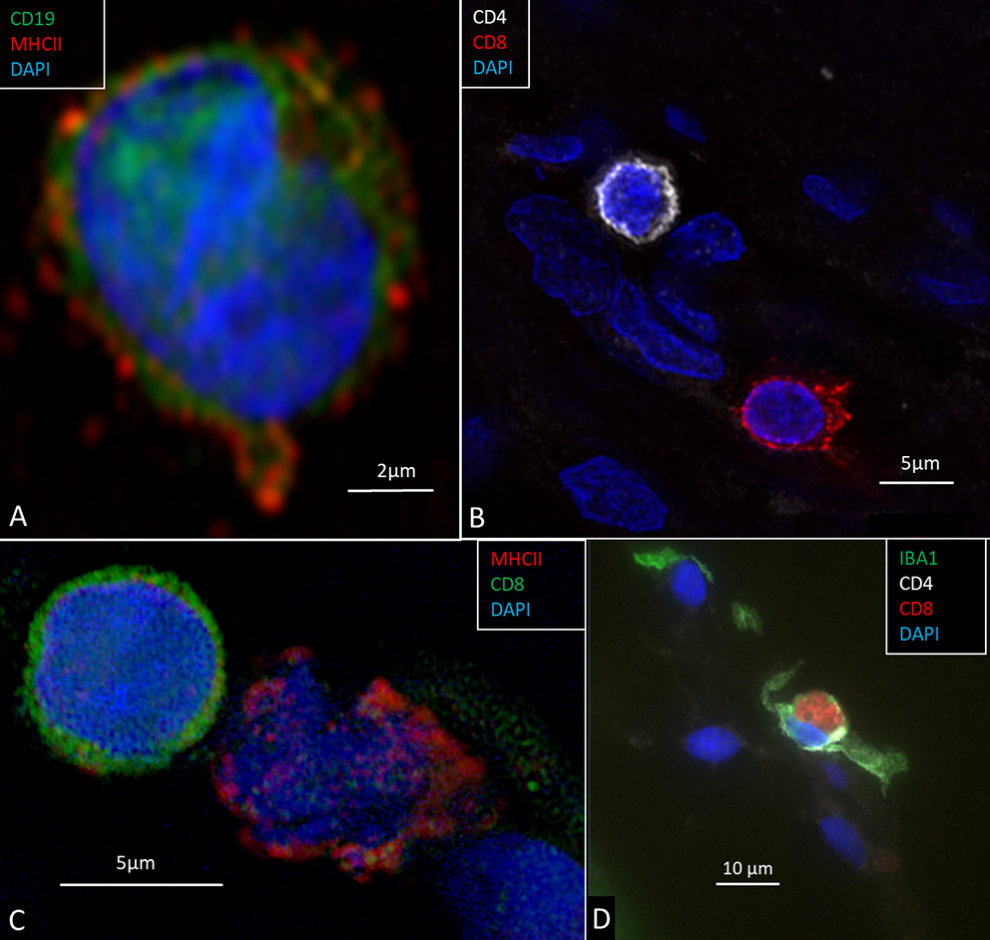
**(A)** CD19-positive B lymphocyte in the modiolus. The cell also expresses MHCII. **(B)** CD4 and CD8-positive T cells in the peripheral region of the RC. **(C)** A CD8-positive lymphocyte and an MHCII-expressing cell in Rosenthal's canal. **(D)** A CD8 cell is seen to physically interact with an IBA1-positive macrophage.

Occasionally, CD4 and CD8 T lymphocytes were observed in the modiolus around blood vessels and along the periphery of Rosenthal's canal (RC) (29). The CD4 and CD8 cells were round or elongated, especially when located at the vessel wall. CD19-positive B cells could be observed expressing MHCII (Figure 13A). The immune cells were mostly located together (Figure 13B) and some CD8 cells lay close to MHCII-expressing macrophages (Figure 13C). Some CD8 cells could be seen to physically interact with IBA1-positive macrophages (Figure 13D). A few isolated CD4 and CD8 cells were identified in the spiral ligament. CD4 and CD8 cells were not found among neurons in RC or in the OC, in the SV, or among neurons.

## Discussion

Most of our knowledge about the microscopic anatomy of the human inner ear is based on paraffin or celloidin-embedded specimens obtained at autopsy. Surgical tissue is difficult to obtain but can have improved preservation due to swift fixation. However, inherent pathology and age can influence outcome, and most often only a small number of tissue specimens are available. The tissue for this study was from patients with meningioma where the cochlea had to be sacrificed, and it was obtained after ethical permission and patient consent. We found that only rarely had the tumor cells infiltrated the cochlea. Meningioma is the most frequent intracranial tumor, constituting around 35% of all cases reported to the Central Brain Tumor Registry of the United States (CBTRUS) (30). After the introduction of the trans-cochlear route to remove large life-threatening petro-clival meningioma at our institute, postoperative complications were drastically reduced, and facial nerve function could be maintained even after tumor elimination medial to the internal auditory canal. A retro-sigmoid craniotomy is often used today for removal of these tumors, but those extending into both the middle and posterior cranial fossae or residuals may best be treated with a trans-petrosal approach, a combination of retro-sigmoid/trans-petrosal approach, or through adjuvant therapy (31).

### Macrophages and Adaptive Immunity

SR-SIM showed many IBA1 positive macrophages in the human cochlea, confirming earlier reports (9). Macrophages are fast-acting cells that play critical roles in mounting innate and adaptive immune responses. They comprise a large volume of cells in the human body (*n* = 10^16^) (15). Their pleomorphic outline may reflect important functional specializations in the human cochlea. Macrophages have pattern-recognition receptors (PRRs) that recognize injury (32, 33) and toll-like receptors (TLRs) that spot infection (34). TLR signaling induces second-line host defense and may be involved in immune responses induced by acoustic injury (35). Thus far, TLR4 has not been identified in the human cochlea, but NF-kB, essential for regulating immune responses, was richly expressed in the spiral ganglion (not shown). Furthermore, IBA1 cells expressed MHCII which is generally not seen unless tissues are specifically challenged (36). Bone-marrow derived cells were found to express MHCII in vestibular end organs and the endolymphatic sac (ES) (37). Lymphocytes were found to be present together with MHCII expressing macrophages in the present study. A few CD4 and CD8 T lymphocytes were observed in the modiolus and RC. Some CD8 cells interacted with macrophages. A few CD4 and CD8 cells were identified in the spiral ligament. It could suggest there is a protective adaptive immune activity in the human spiral ganglion and lateral wall. CD4 and CD8 T-cells may initiate adaptive immune responses from interaction with antigen-presenting macrophages. Antigenic peptide-MHC class II expressed on the surface of antigen-presenting cells provides information to CD4 T helper cells to potentiate adaptive immune responses (38). MHC class II molecules may switch macrophages by upregulation from M2 to M1 macrophages and produce cytokines (13). This may assist antigen presentation to T lymphocytes and cytokine production for further M2 amplification. T cell-induced inflammation may lead to cell injury and neuronal death via pro-inflammatory cytokines and chemokines (39). We found occasional CD19-positive B cells expressing MHCII in the modiolus. These cells can serve as antigen-presenting cells for activation of antigen-specific macrophage-primed CD4 T cells, leading to differentiation into plasma cells (22). MHCII on B cells may also be critical in the pathogenesis of autoimmune conditions in the CNS (23). In a recent investigation, cell types belonging to both the innate and adaptive immune system, including B cells, were found in the cochlea and increased after noise damage, also infiltrating the OC (24). Several genes specific for B and T cells were also expressed in single-cell RNA sequencing analyses. Yet, earlier experiments suggested that inner ear antibodies may derive from the endolymphatic sac (ES) and reach perilymph through the endolymphatic duct (ED), underlining their roles in inner ear immunity (40, 41).

### Macrophages in the OC

Macrophages were observed in the TCL and along lamina fibers near the HP opening. Conceivably, this can be a route for macrophages to migrate into the OC, as has been shown after hair cell injury (42). CD45-positive white blood cells (common leukocyte antigen), derived from the bone marrow, were found to invade cochlear regions susceptible to damage (3). Macrophages may also arrive from the TCL, where IBA1 cells exhibit “antennae”-like processes. Another option is that macrophages are recruited within the sensory organ to repair via activated supporting cells. Expression of immune-related genes was even observed in non-immune epithelial cells in the cochlear sensory epithelium (43). IBA1 cells were found along the perilymph side of the basilar membrane, in the Corti tunnel and below the inner hair cells (IHC) and Hensen cells (9). Similar findings were made in the present investigation. Surprisingly, a few macrophages expressed MHCII, suggesting that they are antigen-presenting cells and can initiate immune activity potentially injurious to the sensory cells. IBA1 cells were recently described near human outer hair cells and dendritic macrophages in the tunnel of Corti (10). Acoustic trauma was found to induce activation of dendritic cells in a reparative process in the OC (1) suggesting a disposal of degeneration products. Here, we found dendritic-like cells in intimate contact with the apical pole of outer hair cells, suggesting ongoing repair and closure of the reticular lamina after hair cell loss. This was seen despite the short time window of SEM analysis. However, whether several hours of temporal bone drilling influenced macrophage activity remains uncertain. Macrophages have also been linked to regeneration of sensory hair cells in the injured avian cochlea (44) but were not verified in a following study. Instead, macrophages were thought to play a role in the preservation of the basilar membrane (45). It was earlier proposed that ectopic IHCs could be a sign of a regenerative capacity that may have been underestimated in man (46). Nonetheless, a similar action and presence of IBA1 cells around IHCs could not be perceived.

### RNA-Scope *in-situ* Hybridization (ISH)

RNA-sequencing techniques are now employed for the study of gene regulation and cell function, including sensory organs. Cell-specific regulatory networks can be monitored to better understand their functions and how they may be influenced by noxious factors and treated (47). RNAscope^®^ ISH (48, 49) is a highly sensitive technique that allows identification and quantification of different mRNAs in various subsets of the inner ear (50). In this study, *CX3CL1* gene expression was demonstrated for the first time in the human cochlea. Swift fixation maintained mRNA and tissue integrity for proper detection. The methods we used allowed quantitative analysis of RNA-scope puncta through ImageJ software, but unbiased quantification of dots was difficult due to the limited amount of human tissue. *GJB6*, encoding Connexín30 protein (Cx30) was used as an additional control gene since it is expressed in selected cell strata of the SV (28).

### Fractalkine Signaling in the Human Cochlea

Migration of tissue macrophages and adherence to cells is monitored by chemokine signaling (51). In mature animals, cochlear macrophages express CX3CR1 (Supplementary Figure 1) acting as a chemotactic receptor and adhesion molecule when coupled to its ligand CX3CL1 (42). FE-SEM, TEM, confocal, and super-resolution immunohistochemistry showed in the present study that many earlier unidentified cells in the human cochlea may represent macrophages. Macrophages show signs of locomotion and migration similar to brain microglia. The cells are endowed with projecting micro-filaments or “antennae” (width 0.1–0.2 μm) pointing at or adhering to surrounding cells. Macrophages can reach minor crevices and perforate basal laminae and tight junctions crossing cell barriers. The micro-projections may serve as “tracking dogs” and are difficult to envisage by regular immunofluorescence but are recognized with SR-SIM and TEM. If they enter, the HP cannot be confirmed. The long IBA1 processes accompany nerve fibers and terminate on their surfaces as synapse-like knobs. The axonal initial segments (AISs) are often endowed with contacts, and adhesion seems to be facilitated by the lack of myelin. The SR-SIM suggests that they even penetrate into the ganglion cell soma in direct contact with the CX3CL1-expressing cell soma membrane (Figure 9B). IBA1 cells also swarm around myelinated axons, and their process may reach the perineural space, possibly also the Ranvier nodes. This indicates that spiral ganglion neurons may be incorporated by macrophages into a wider neuro-immune system.

Few *CX3CL1* gene transcripts were found in the lateral wall and were mostly restricted to vascular endothelial cells or cells surrounding the capillaries (Supplementary Figures 1A–C). This contrasted with the wide expression of *GJB6* gene transcripts in the SV, except the marginal cells, which were devoid of *GJB6* gene puncta (Figure 12A). *CX3CL1* genes were noticed in the supporting cells in the OC. There were abundant gene puncta in the spiral ganglion and certain Schwann cells, explaining the enrichment of IBA1 cells (29). The large number of macrophages in the human spiral ganglion indicates a particular role for human auditory nerve homeostasis. In the brain, microglial chemokine receptors influence nerve growth (52) and can increase secretion of neuroprotective brain-derived neurotrophic factor (BDNF) (53). Cells can act to restore tissue and improve cell regeneration (54–57). Fractalkine signaling was found to regulate macrophage recruitment into the cochlea and promoted survival of spiral ganglion neurons after selective hair cell lesion (16). Also lack of fractalkine receptor on macrophages impaired recovery of ribbon synapses after noise trauma (17). Animals displayed increased synaptic degeneration and deterioration of neural responses. This indicates that macrophages can stimulate repair of injured synapses and depends on fractalkine signaling. Macrophage neuroprotection could explain the human auditory nerve's exceptional resistance to degeneration following hair cell loss.

### Macrophages and Inner Ear Immune Regulation

**Figure 14:**
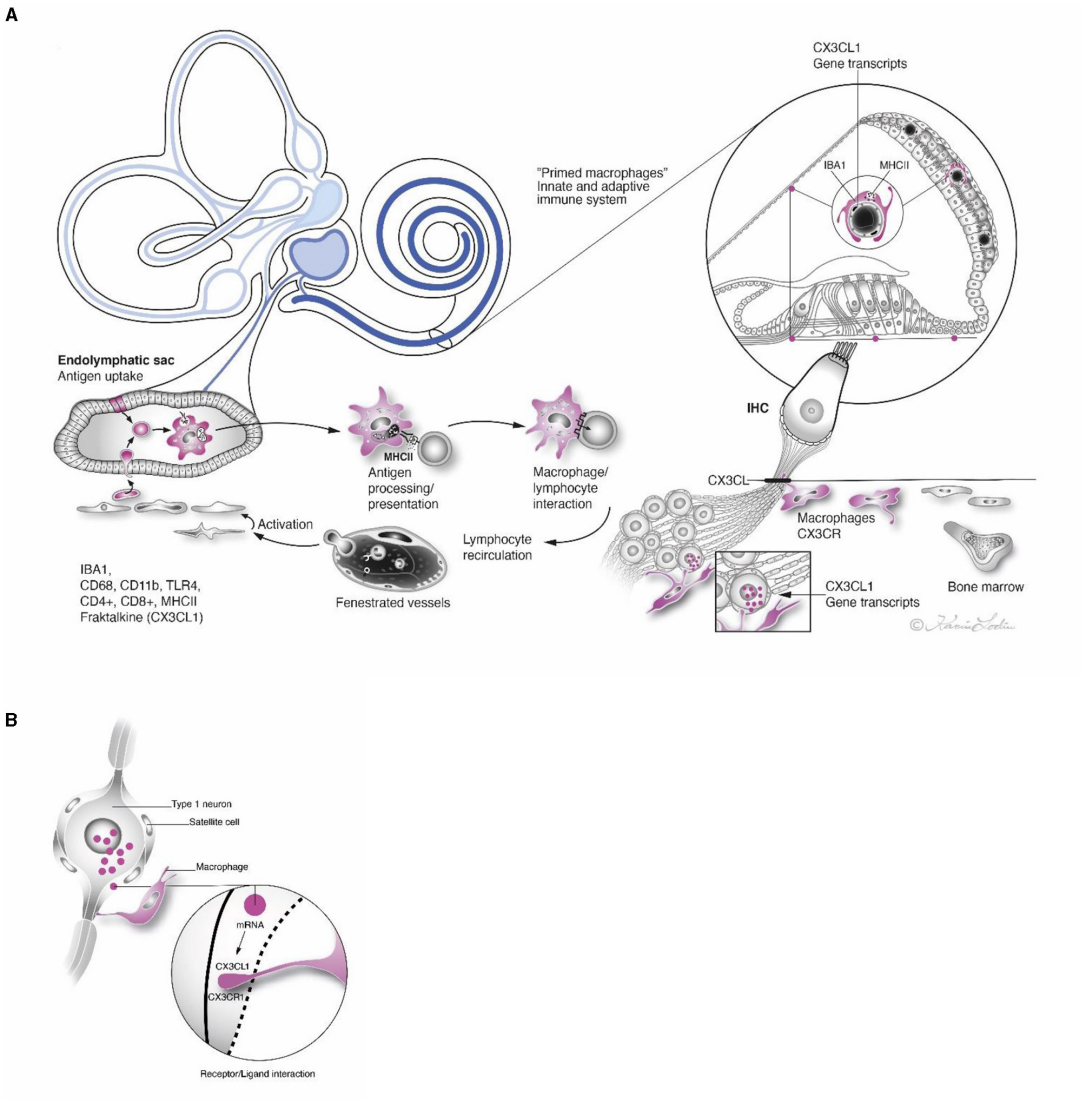
**(A)** Antigen entering the inner ear from infection-prone areas may be thrust through the endolymphatic duct to the ES. The sac contains bone marrow-derived immune competent cells that may engulf and immune process agents with the help of MHCII and TLR4. These antigen-presenting cells may “prime” memory cells that reenter the inner ear via the circulation and form protective barricades in different regions. Strategic expressions of *CX3CL1* gene transcripts are outlined. **(B)** Framed region in **(A)** show receptor/ligand interaction in satellite cell in higher magnification.

The human inner ear is a protected by a fluid-filled enclave earlier believed to be relatively isolated from the general immune system. Recent data have shown that it contains resident macrophages that are readily exchanged through a population of heterogeneous immune cells derived from the bone marrow (3, 58). A surprising interaction between local and systemic inflammation was revealed by lipopolysaccharide and ototoxic synergism (59). Therefore, the inner ear may be more influenced by inflammatory activity in the rest of the body than earlier believed. Macrophages may protect the ear from nearby infection-prone areas by quickly recruiting “inflammatory” CCR2^+^ monocytes which can also lead to injury. One way to evade damage is the transfer of infectious material along the ED to the ES for disposal and immune processing. The ES contains a multitude of macrophages co-expressing MHCII and TLR4-positive cells. Some epithelial cells represent “fixed” macrophages that may loosen and migrate. After primary antigen processing, programmed memory cells may reenter the cochlea and auditory nerve. The “primed” cells could recirculate and target locations in the inner ear by chemokine signaling (Figures 14A,B). Additional evidence for the role of the ES in inner ear immunity was recently reached (60). Physically interacting immune cells and MHCII molecular aggregates appeared in a large number of macrophages and even epithelial cells in the ES. Organelles, plasma membranes, and multi-vesicular bodies expressed MHCII, suggesting antigen proteolysis and peptide coupling to the MHCII complex.

The introduction of foreign substances and cochlear prosthesis into the inner ear could activate the local immune system. Phagocytosing macrophages and lymphocytes were found histologically around the electrode, within cochlear vessels, and even the vestibular organ following implantation (61–63). The cells may be involved in wound healing, but remarkably, spiral ganglion cells were found to be “wrapped” by ramified macrophages with long processes contacting both satellite cells and ganglion cells (61). Macrophages physically interacted with neighboring cells, substantiating a surveillance function. Intercellular membrane specializations were perceived between macrophages and neurons by TEM (11, 60). Chemokines signaling may enhance target precision and molecular interaction. This suggests that the IBA1 cells may physically protect or even engulf neural cell bodies. Nevertheless, strategies to modulate their immune-regulatory activity and exchange across the semi-immune-privileged blood-labyrinth barrier (64) may hold promise in cases of suspected dysregulation and immune disease. Preservation of the acoustic nerve after hair cell loss is indispensable for the successful outcome of cochlear implantation. Our SEM demonstrated that cells occasionally associated with degradation of outer hair cells. We believe that these cells represent dendritic cells or macrophages. Whether these cells are involved in auto-aggressive self-targeting action or cellular maintenance remains unclear but is of paramount importance to determine.

## Author Contributions

WL performed CM, SR-SIM and RNAscope^®^ analyses in Uppsala. AS-F, RG, and HR-A performed SEM and TEM analyses of human cochleae at Innsbruck University and Uppsala. HR-A was the head of the laboratory in Uppsala and planned the project, analyzed the images together with WL, and wrote the manuscript. AS-F was the head of the laboratory in Innsbruck. ND-L performed the surgery. All authors contributed to the article and approved the submitted version.

## Funding

This study was supported by the Swedish Research Council [2017-03801], the Tysta Skolan Foundation, the Swedish hearing research foundation (hrf), and generous private funds from Arne Sundström, Sweden. The project was supported by MED-EL, Innsbruck, Austria under an agreement and contract with Uppsala University. The funder had no role in study design, data collection and analysis, decision to publish, or preparation of the manuscript. This research has also been financially supported by the K-regio project eVITA (electrical Vestibular Implant Tirol Austria) sponsored by EFRE (Dieses Projekt wird aus Mitteln des Europäischen Fonds für regionale Entwicklung kofinanziert, http://www.efre.gv.at/, accessed August 1, 2021). The Austrian Science Fund (FWF Austria) projects I 4811 (Neurotrophins in developing human inner ear and in HNSCC) & project I 4147-B (Modeling electrical stimulation of the human cochlear nerve) and FWF project I 3154-B27 (-Gapless Man: Machine Interface) also supported this research.

## Conflict of Interest

The authors declare that the research was conducted in the absence of any commercial or financial relationships that could be construed as a potential conflict of interest.

## Publisher's Note

All claims expressed in this article are solely those of the authors and do not necessarily represent those of their affiliated organizations, or those of the publisher, the editors and the reviewers. Any product that may be evaluated in this article, or claim that may be made by its manufacturer, is not guaranteed or endorsed by the publisher.

## Supplementary Material

Supplementary Figure 1 RNA-scope ISH and SR-SIM of *CX3CL1* gene transcripts in the lateral wall of the cochlea **(A–C)** and the spiral lamina region **(D–F)**. Framed area in **A** is magnified in **B** and shows a spiral prominence capillary with endothelial cells expressing *CX3CL1* gene transcripts in cell nuclei. There are few genes in other cell nuclei including outer sulcus epithelium. **(C)** Endothelial cell nuclei of a capillary in the SV contain gene transcripts. **(D)** Spiral lamina nerve fibers are shown in lower left with few gene puncta in surrounding Schwann cells. Some cells in the TCL contain numerous gene puncta (upper right). **(E)** Epithelial cells facing the endolymph in the inner sulcus contain gene puncta. **(F)** Gene transcripts located in cell nuclei in the spiral lamina. MNF; myelinated nerve fibers.

Supplementary Figure 2 Confocal microscopy of fractalkine receptor (CX3CLR) expression in the human spiral ganglion. **(A)** Macrophages express both CX3CLR and MHCII. **(B)** Inset shows framed cell in higher magnification. **(C)** Macrophages express MHCII in spiral ganglion. **(D)** Same section as C shows that macrophages co-express MHCII and fractalkine receptor.

## Abbreviations

AIS: axonal initial segment
BM: basilar membrane
Cx30: connexin30
DAPI: 4′, 6-diamidino-2-phenylindole dihydro-chloride
ES: Endolymphatic sac
ED: Endolymphatic duct
EDTA: ethylene-diamine-tetra-acetic acid
GFAP: glia fibrillary acidic protein
GJ: gap junction; Gap junction gene *GJB6* encodes connexin30 protein
HP: habenula perforata
LW: lateral wall; fractalkine protein (CXC3CL) is a chemokine (C-X3-C motif) ligand 1 encoded by the *CX3CL1* gene; fractalkine receptor protein (CX3CR1) is a CX3C chemokine receptor 1 in humans is encoded by the *CX3CR1* gene.
IHC: inner hair cell
CM: confocal microscopy
IBA1: ionized calcium-binding adaptor molecule 1
TMEM119: transmembrane protein 119 (marker of microglia in human brain)
P2Y12: chemoreceptor for adenosine diphosphate (expressed on microglia)
CD: cluster of differentiation
MHCII: major histocompatibility complex type II
CD4: cluster of differentiation 4 (a receptor found on the surface of immune cells such as T helper cells, monocytes, macrophages, and dendritic cells).
CD8: cluster if differentiation 8 (a co-receptor expressed on cytotoxic T cells, natural killer cells and dendritic cells); CD68 is a protein expressed by tissue macrophages; CD11b
IHC: inner hair cell; Integrin alpha M macrophage-1 antigen is expressed on leukocytes involved in the innate immune system
OC: organ of Corti
OHC: outer hair cell
RC: Rosenthal's canal
SEM: scanning electron microscopy
RNA scope: ribonucleic acid detection device
SGCs: satellite glia cells
SGN: spiral ganglion neuron
SR-SIM: super-resolution structured illumination microscopy
SP: spiral prominence
SV: stria vascularis
TEM: transmission electron microscopy
TUJ1: tubulin-1
Type I cells: large spiral ganglion neurons (~95% of total numbers)
Type II cells: small spiral ganglion neurons (~5% of total numbers)
TLR4: toll-like receptor family 4 belong to the pattern recognition receptor family activating innate immunity
TCL: tympanic covering layer.
