# Supplementary figures and images for: Distribution of Immune Cells Including Macrophages in the Human Cochlea
Source: Front Neurol. 2021 Nov 22;12:781702. doi: 10.3389/fneur.2021.781702 (PMC8645652; doi:10.3389/fneur.2021.781702)

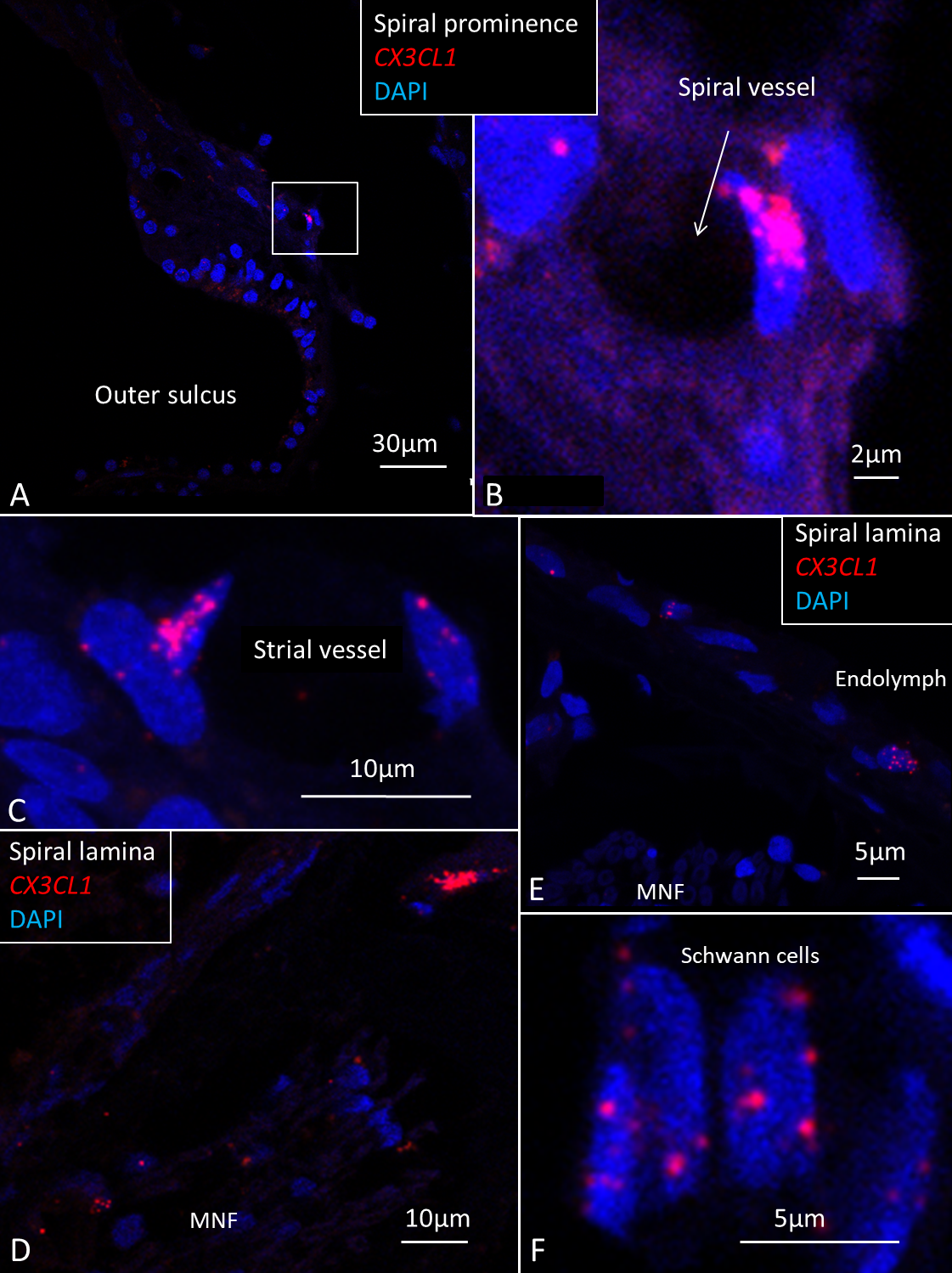

Supplement: Supplementary Figure 1 — RNA-scope ISH and SR-SIM of CX3CL1 gene transcripts in the lateral wall of the cochlea (A–C) and the spiral lamina region (D–F). Framed area in A is magnified in B and shows a spiral prominence capillary with endothelial cells expressing CX3CL1 gene transcripts in cell nuclei. There are few genes in other cell nuclei including outer sulcus epithelium. (C) Endothelial cell nuclei of a capillary in the SV contain gene transcripts. (D) Spiral lamina nerve fibers are shown in lower left with few gene puncta in surrounding Schwann cells. Some cells in the TCL contain numerous gene puncta (upper right). (E) Epithelial cells facing the endolymph in the inner sulcus contain gene puncta. (F) Gene transcripts located in cell nuclei in the spiral lamina. MNF; myelinated nerve fibers. [file Image_1.TIFF]

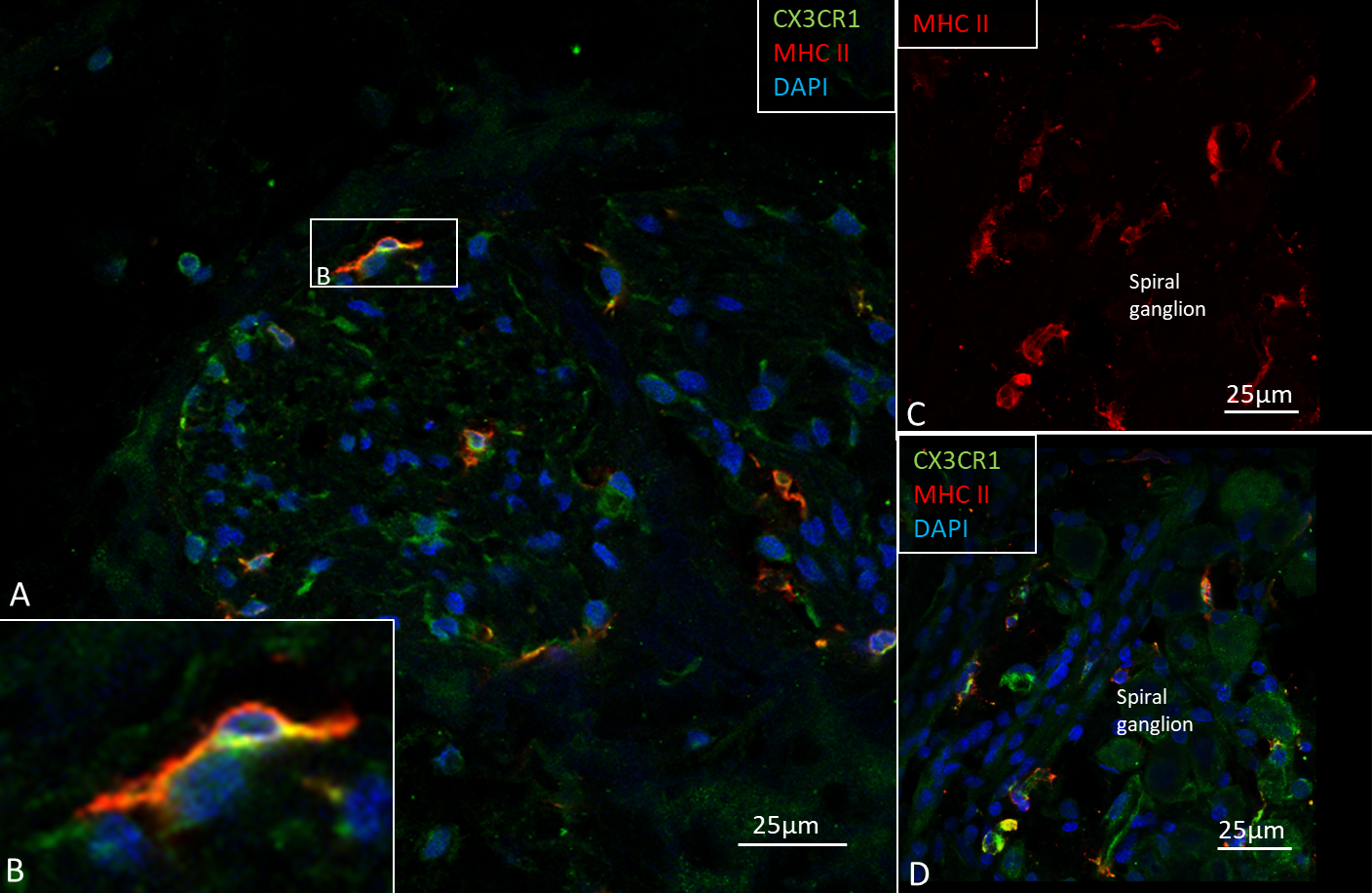

Supplement: Supplementary Figure 2 — Confocal microscopy of fractalkine receptor (CX3CLR) expression in the human spiral ganglion. (A) Macrophages express both CX3CLR and MHCII. (B) Inset shows framed cell in higher magnification. (C) Macrophages express MHCII in spiral ganglion. (D) Same section as C shows that macrophages co-express MHCII and fractalkine receptor. [file Image_2.tiff]
